# Glioma subtype classification from histopathological images using in-domain and out-of-domain transfer learning: An experimental study

**DOI:** 10.1016/j.heliyon.2024.e27515

**Published:** 2024-03-06

**Authors:** Vladimir Despotovic, Sang-Yoon Kim, Ann-Christin Hau, Aliaksandra Kakoichankava, Gilbert Georg Klamminger, Felix Bruno Kleine Borgmann, Katrin B.M. Frauenknecht, Michel Mittelbronn, Petr V. Nazarov

**Affiliations:** a Bioinformatics Platform, Department of Medical Informatics, Luxembourg Institute of Health, Strassen, Luxembourg; b Dr. Senckenberg Institute of Neurooncology, University Hospital Frankfurt, Frankfurt am Main, Germany; c Edinger Institute, Institute of Neurology, Goethe University, Frankfurt am Main, Germany; d Frankfurt Cancer Institute, Goethe University, Frankfurt am Main, Germany; e University Cancer Center Frankfurt, Frankfurt am Main, Germany; f University Hospital, Goethe University, Frankfurt am Main, Germany; g Laboratoire national de santé, National Center of Pathology, Dudelange, Luxembourg; h Multi-Omics Data Science group, Department of Cancer Research, Luxembourg Institute of Health, Strassen, Luxembourg; i Luxembourg Centre of Neuropathology, Dudelange, Luxembourg; j Klinik für Frauenheilkunde, Geburtshilfe und Reproduktionsmedizin, Saarland University, Homburg, Germany; k Department of Cancer Research, Luxembourg Institute of Health, Strassen, Luxembourg; l Luxembourg Centre for Systems Biomedicine, University of Luxembourg, Belval, Luxembourg; m Department of Life Sciences and Medicine, University of Luxembourg, Esch-sur-Alzette, Luxembourg; n Faculty of Science, Technology and Medicine, University of Luxembourg, Esch-sur-Alzette, Luxembourg; o Haupitaux Robert Schumann, Kirchberg, Luxembourg

**Keywords:** Digital pathology, Whole slide images, Glioma, Deep learning, Transfer learning

## Abstract

We provide in this paper a comprehensive comparison of various transfer learning strategies and deep learning architectures for computer-aided classification of adult-type diffuse gliomas. We evaluate the generalizability of out-of-domain ImageNet representations for a target domain of histopathological images, and study the impact of in-domain adaptation using self-supervised and multi-task learning approaches for pretraining the models using the medium-to-large scale datasets of histopathological images. A semi-supervised learning approach is furthermore proposed, where the fine-tuned models are utilized to predict the labels of unannotated regions of the whole slide images (WSI). The models are subsequently retrained using the ground-truth labels and weak labels determined in the previous step, providing superior performance in comparison to standard in-domain transfer learning with balanced accuracy of 96.91% and F1-score 97.07%, and minimizing the pathologist's efforts for annotation. Finally, we provide a visualization tool working at WSI level which generates heatmaps that highlight tumor areas; thus, providing insights to pathologists concerning the most informative parts of the WSI.

## 1 Introduction

Diffuse gliomas are the most common type of brain tumors in adults with up to 80% of primary malignant central nervous system (CNS) tumors [1]. The last $5^{th}$ edition of the World Health Organization (WHO) classification of CNS tumors released in 2021 proposes a refinement of the classification of adult-type diffuse gliomas based on molecular profiles, largely dependent on isocitrate dehydrogenase 1 or 2 (IDH1/2) mutation status and 1p/19q codeletion status, resulting in 3 primary tumor subtypes: IDH-mutant, 1p/19q codeleted oligodendroglioma; IDH-mutant astrocytoma; and IDH-wildtype glioblastoma [2]. However, the combination of molecular information and histopathological features extracted from the Whole Slide Images (WSI) remains the gold standard for diagnosing and grading CNS tumors [3]. Correct classification of diffuse glioma subtypes is of utmost importance, given that treatment and patient survival largely depend on the tumor subtype, with low-grade gliomas reaching 5-year survival rates up to 80%, and high-grade gliomas below 5% [4].

Since the diagnosis is based on subjective pathologist assessment, prone to inter- and intra-observer variation, computer-aided WSI analysis is essential to improve the reproducibility and diagnostic accuracy, and reduce the workload of pathologists [5], [6]. Computer-aided image analysis has been recently used for the classification of tumor subtypes [7], [8] or grading [9], [10], [11] of gliomas, or for survival prediction [12] from digital histopathological images. Closely related to this task is the prediction of IDH1/2 mutation status, as an important diagnostic and prognostic biomarker in diffuse gliomas [13]. Other methods propose integrating features extracted from histopathological images with molecular features [14], [15], or combining WSIs with Magnetic Resonance Imaging (MRI) [16], [17].

These approaches dominantly use Convolutional Neural Networks (CNN) for extracting features from WSIs, either trained from scratch on the histopathological image dataset of interest [10], [13], [17]; or using the transfer learning techniques with models pretrained in a domain of natural images [7], [15]. Although CNNs are still considered to be state-of-the-art models for image classification, a change of paradigm can be recently observed towards the use of attention-based architectures and transformer networks in computer vision, challenging the superiority of CNNs. These can be either hybrid architectures where CNNs are augmented with an attention mechanism to capture long-range dependencies; thus, alleviating the major limitation of CNNs which only operate locally [18]; or pure attention-based transformer models (i.e. Vision Transformers (ViT)) [19] which can achieve performance comparable to CNNs (or even outperform them on some tasks) without using convolutions, but typically require more training data. Recently introduced Data-efficient image Transformers (DeiT) have shown that with improved data augmentation and regularization strategies, ViTs can be trained with fewer data without any significant changes in architecture [20].

This trend can be also observed in digital pathology. A breast cancer classification model based on color deconvolution and transformer architecture is proposed in [21]. ViT for tumor detection in sentinel lymph nodes, diffuse large B-cell lymphoma, breast, and lung adenocarcinoma show comparable performance to ResNet50 model [22]. Hierarchical pyramid ViT architecture leverages a hierarchical structure of morphological features at different image resolutions, starting from 16×16 images capturing information at the cell level, 256×256 images capturing cell-cell interactions and 4096×4096 images representing the cell clusters in tissue micro-environment [23].

Pretraining the models on datasets of natural images, where large-scale datasets (e.g. ImageNet [24]) are publicly available and models can be pretrained in a supervised fashion, is common in digital pathology [22]. We refer to this in the remaining text as the out-of-domain pretraining. However, it was shown that when the source and the target domains are not well matched, which certainly is the case when ImageNet pretraining is used in a downstream histopathological domain task, transferability reduces [25]. Therefore, efforts were made to use in-domain pretraining using the publicly available annotated datasets of histopathological images, such as Camelyon16, and then transfer the model parameters to another digital pathology task [26], [27]. However, Camelyon16 is a relatively small dataset, and the main benefit of transfer learning is observed when models are pretrained on large-scale data.

In the absence of large-scale datasets of histopathological images annotated at the pixel or tile level, but with the availability of WSIs with known patient diagnoses, self-supervised learning approaches are introduced as an alternative [23], [28], [29], [30], [31]. The idea is to define a domain-specific pretext task that does not require exhaustive annotations by pathologists, where labels are inherent in the source data (e.g. magnification prediction, hematoxylin channel prediction [32], or cross-stain prediction [33]). Different self-supervised approaches have been proposed in digital pathology, including contrastive self-supervised learning (SimCLR) [30], semantically-relevant contrastive learning (SRCL) [29], or Bootstrap Your Own Latent (BYOL) [28]. Another approach is to use multi-task pretraining by defining a set of classification tasks for multiple low- and middle-scale histopathological datasets, and training a model that will minimize loss aggregated over all tasks [31].

In this paper, we provide a comprehensive comparison of various in-domain and out-of-domain transfer learning approaches for the classification of adult-type diffuse gliomas from WSIs, and highlight their major advantages and disadvantages. Recent attempts indicate that in-domain transfer learning may outperform out-of-domain pretraining [23], [28], [29], [30], [31]; however, only limited efforts were undertaken so far to quantify the benefits of in-domain transfer learning in digital pathology in a systematic way. Existing studies were mostly focused on the domain of radiology (MRI), measuring the effect of different transfer learning strategies for the object detection task [34].

To further improve the model performance, we propose a two-step semi-supervised learning approach: the pretrained model from the previous step is used to predict the pseudo labels of the unannotated tiles in WSIs. The model is subsequently retrained using the ground-truth tiles labeled by the pathologists, augmented with the pseudo-labeled ones annotated by the model; thus, minimizing the efforts for annotation by the trained pathologist.

Finally, the model at the output of step 2 of semi-supervised learning is used to aggregate the predictions from the tile level to the slide level. The predicted classes are overlaid on the WSI as a heatmap, emphasizing and drawing the pathologist's attention to the most informative areas in the image corresponding to tumor tissue, normal brain tissue or necrosis. Furthermore, we present a new dataset for diffuse glioma subtype classification, annotated by experienced pathologists at the tile level, which we use for fine-tuning and evaluation of models.

## 2 Material and methods

**Figure 1:**
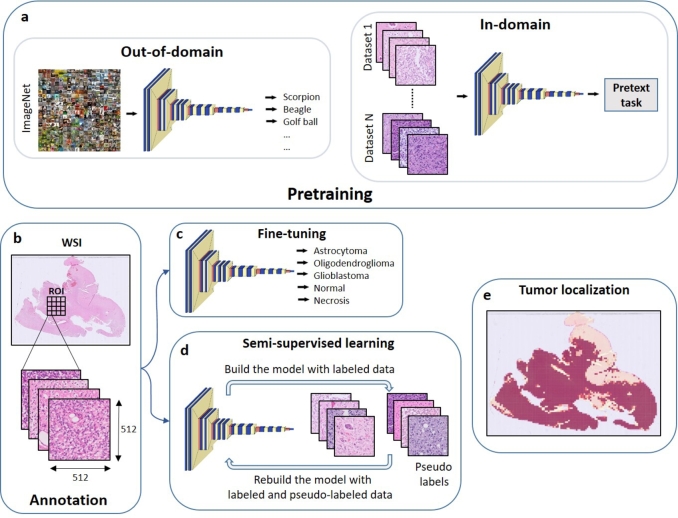
Architecture of the proposed computer-aided glioma subtype classification system from histopathological images. (a) In out-of-domain pretraining models are pretrained on large scale natural image data, whereas for in-domain pretraining collections of multiple public histopathological image datasets are used to pretrain the models in a self-supervised fashion. (b) Original whole-slide image (WSI) is annotated by a trained pathologist to define the region of interest (ROI) and divide it into 512 × 512 pixels tiles. (c) Pretrained model is fine-tuned in a supervised fashion using the annotated in-house glioma dataset and directly used to predict glioma subtypes. (d) Semi-supervised learning step is added to predict the pseudo labels of the tiles outside of ROI, and rebuild the model using the ground -truth labels and pseudo labels. (e) The tile level predictions are aggregated at the WSI level as a heatmap to localize the areas with the tumor tissue.

The block diagram of the proposed computer-aided glioma subtype classification system from histopathological images is shown in Fig. 1.

### 2.1 Dataset and study design

The dataset contains 75 H&E stained WSIs of 29 adult-type diffuse glioma cases collected at the National Center of Pathology (NCP), Luxembourg National Health Laboratory (Laboratoire national de santé - LNS) during the years 2017–2021. WSIs were acquired with an IntelliSite Ultra Fast digital slide scanner from Philips containing a 20x/0.75 NA Plan Apo objective with an average slide resolution of 0.25um/pixel.

Neuropathological diagnostics of tumor samples (histology, immunohistochemistry, epigenetic or genetic analysis) was performed by a board-certified neuropathologist (MM) according to the $5^{th}$ edition of the WHO classification of CNS tumors [2] and only pseudonymized, region-annotated digital images were exchanged for bioinformatic processing. Three primary tumor subtypes are classified into IDH-mutant, 1p/19q codeleted oligodendroglioma; IDH-mutant astrocytoma; and IDH-wildtype glioblastoma.

**Tissue Processing, Staining, Imaging and Export**: Patient tissues were routinely fixed directly after surgery in 4% neutral buffered formalin, gradually dehydrated and cleared with an automatic tissue processor, followed by paraffin embedding (formalin-fixed paraffin embedded - FFPE). FFPE tissue blocks were sectioned with a microtome at 3μm and 7μm thickness, placed on a glass slide, stained with H&E and scanned with the Philips IntelliSite Ultra Fast scanner. Images were exported as big-tiff images with the following settings: scan factor 20 and quality 80 or 100. Region annotation of WSIs was done by a board-certified pathologist (MM) using the Aperio image scope version 12.3.3 software.

The detailed statistics of the dataset is given in Table 1. The dataset is dominated by male participants (65.5%), which correlates with findings that gliomas are 50% more prevalent in males than in females [35]. Grade 4 IDH-wildtype glioblastoma, as the most common malignant primary brain tumor in adults [36], is also the most prevalent in our dataset (41.4%). IDH-mutant astrocytoma is represented by 27.6% of WSIs, out of which 25% are grade 2, 62.5% are grade 3 and 12.5% are grade 4 CNS tumors. IDH-mutant, 1p/19q co-deleted oligodendroglioma is represented by 27.6% of WSIs, out of which 50% are grade 2, and 50% are grade 3 CNS tumors. The brain WSIs of a non-cancer patient were used as normal controls.

**Table 1:** Statistics of the in-house dataset.

**Participants**				
Gender	Male	Female	Total	
Number of participants	19	10	29	
Age	57.3 (18.5)	49.2 (13.3)	54.5 (17.3)	

IDH-mutant, 1p/19q codeleted (odg*)	4	4	8	
IDH-mutant (ac**)	4	4	8	
IDH-wildtype (gbm***)	11	1	12	
Normal brain tissue	0	1	1	

**CNS WHO grade**	2	3	4	NA

IDH-mutant, 1p/19q codeleted (odg*)	4	4	0	0
IDH-mutant (ac**)	2	5	1	0
IDH-wildtype (gbm***)	0	0	12	0
Normal brain tissue	0	0	0	1

*odg: oligodendroglioma; **ac: astrocytoma; ***gbm: glioblastoma

### 2.2 Data preprocessing

Given the extremely large resolution of WSIs of typically 100000×100000 pixels, they are processed in patches or tiles. Regions of interest (ROI) were annotated by marking several rectangular areas in the WSIs (Fig. 1b), as different levels for normal brain tissue (white and gray matter), necrosis, and the respective tumor entity, e.g. IDH-mutant, 1p/19q codeleted oligodendroglioma; IDH-mutant astrocytoma; and IDH-wildtype glioblastoma. These ROIs are further divided into square 50% overlapping 512×512 tiles, each of them associated with a particular class.

Image augmentation is applied to all extracted tiles in the training dataset by flipping and rotating by $90^{\circ }$, $180^{\circ }$ and $270^{\circ }$, leading to 8 augmented views of each tile. No augmentation is applied to test data. Basic statistics of the training and test subsets is provided in Table 2. Note that the training dataset is highly imbalanced with the normal brain class represented with 18 times more images than the necrosis class. This has to be taken into account during the model training and evaluation.

**Table 2:** Tile level statistics (after image augmentation) of the training and test subsets.

**Class**	**Training**	**Test**
IDH-mutant, non-codeleted (astrocytoma)	30040	465
IDH-mutant, 1p/19q codeleted (oligodendroglioma)	27072	431
IDH-wildtype (glioblastoma)	13064	241
Normal brain tissue	56291	2947
Necrosis	3112	90

**Total**	129579	4174

### 2.3 Transfer learning

We investigate state-of-the-art transfer learning strategies for computer-aided glioma subtype classification, including supervised out-of-domain transfer learning, where models are pretrained on a large collection of natural images; and in-domain transfer learning, where models are pretrained on medium-to-large-scale publicly available collections of histopathological images (Fig. 1a).

#### 2.3.1 Out-of-domain transfer learning

A variety of CNN-based models (VGG16, VGG19 [37], ResNet18, ResNet50 [38], InceptionV3 [39], MobileNetV2 [40], DenseNet121 [41]) pretrained on ImageNet dataset that contains over 1.2 million of natural images was used for out-of-domain transfer learning. Following the latest trends in image classification, we also experiment with Transformer models, i.e. Vision Transformers (ViT) [19], and Swin transformers [42]. Finally, we use the hybrid CNN-Transformer model, where the input sequence for ViT is generated from the feature maps of ResNet50 network [19]. The hybrid model can capitalize on the complementary strengths of CNN and transformer architectures, with ResNet50 capturing details within the local image regions, and ViT enhancing the model's ability to capture the global contextual information from images.

Two strategies for training the models are applied, i.e. training from scratch, and pretraining followed by fine-tuning. In training from scratch, a model with randomly initialized weights is trained directly on the target in-house dataset. In fine-tuning, weights and biases of the pretrained model are used to initialize the network, which is then retrained on the target in-house dataset, meaning that all network parameters are updated.

Details about the pretrained models, including model size, number of model parameters and topological network depth (number of layers in a neural network) are shown in Table 3. Note that only the layers with trainable weights are considered for calculating the network depth.

**Table 3:** Deep learning architectures used for out-of-domain transfer learning.

**Model**	**Size [MB]**	**Number of parameters**	**Depth**
VGG16	528	138.4M	16
VGG19	548	143.7M	19
ResNet18	45	11.7M	18
ResNet50	97	25.6M	50
InceptionV3	91	23.8M	95
MobileNetV2	14	3.5M	53
DenseNet121	31	8M	121
ViT	330	86.4M	38
Swin-T	108	28.3M	53
ResNet50-ViT	438	114.5M	102

#### 2.3.2 In-domain transfer learning

Two strategies are investigated for in-domain transfer learning, i.e. self-supervised and multi-task learning.

We employ a contrastive self-supervised learning strategy, as given in [30], where two stochastically augmented versions of the same tile are created and the model parameters are optimized to maximize the similarity between representations of these two versions of the tile (positive examples). At the same time, dissimilarity to all other tiles in the batch (negative examples) is emphasized using the contrastive NT-Xent loss function. ResNet18 was used as the backbone network to extract the features. The model was pretrained using a collection of 57 histopathological image datasets originating from 22 organs (including The Cancer Genome Atlas Program (TCGA^1^) and Clinical Proteomic Tumor Analysis Consortium (CPTAC), as well as multiple publicly available challenge datasets). Different staining methods (H&E, Wright's stain, Anti CD3, CD8, Jenner–Giemsa, H-DAB, PAS) and various magnification levels (10×, 20×, 40×, 100×) were used across datasets. For additional information about the datasets, the reader is referred to [30].

The second self-supervised learning approach named TransPath [28] employs the Bootstrap Your Own Latent (BYOL) strategy, which does not require negative examples. Two networks with identical architectures, but different weights, i.e. online network and target network, were pretrained using two augmented views of each tile. A hybrid model with ResNet50 as a local feature pre-extractor and ViT that learns global features is used as a backbone network. Models were pretrained using a collection of 32529 WSIs and 2.7 million randomly selected tiles originating from TCGA and Pathology AI Platform (PAIP^2^), covering 25 anatomic sites and 32 cancer subtypes.

CTransPath [29] uses a contrastive learning approach built on top of MoCo v3 [43], but redefines positive examples in the self-supervised learning task, i.e. in addition to an augmented view of the input instance, pseudo-positive semantically relevant examples are selected from a memory bank; thus, increasing the diversity of positives. A hybrid model with a three-layer CNN as a local feature pre-extractor and Swin Transformer that learns global features is used as a backbone network. The same dataset was used for pretraining as in [28], but containing all tiles, instead of approximately 100 randomly selected tiles from each WSI, leading to a largest available dataset so far composed of more than 15 million of tiles.

Another model uses multi-task learning strategy, where the same network architecture is shared across multiple classification tasks that correspond to multiple histopathological image datasets, and a separate classification head is attached to each task [31]. Once the per-task categorical cross-entropy loss is calculated for each task, these losses are aggregated, and the network parameters are optimized to minimize the average total loss. Two CNN architectures are used as a backbone network: ResNet50 and DenseNet121. Models were pretrained on a collection of 22 histopathological datasets for both binary and multi-class classification tasks containing 882800 images in total. Different staining methods were used across datasets, including H&E, Diff-Quik, May-Grunwald-Giemsa and Masson's trichrome staining. For additional information about the datasets the reader is referred to [31].

The pretrained network parameters were further transferred for fine-tuning using the in-house dataset (Fig. 1c). Two fully connected layers are added on top of the backbone network, followed by dropout layers to prevent overfitting. The details about the models for in-domain transfer learning are provided in Table 4.

**Table 4:** Deep learning architectures used for in-domain transfer learning.

**Pretraining**	**Model**	**Size [MB]**	**Parameters**	**Depth**
Self-supervised	SimCLR (ResNet18)	45	11.7M	18
	BYOL (ResNet50+ViT)	377	98.8M	418
	MoCo v3 (CNN+Swin-T)	105	27.5M	223

Multi-task	ResNet50	97	25.6M	50
	DenseNet121	31	8M	365

#### 2.3.3 Semi-supervised transfer learning

We further propose a semi-supervised learning approach, where the fine-tuned models presented in Section 3.2 are used to predict the pseudo labels of the unannotated tiles in WSIs (i.e. the ones not belonging to ROIs). The model is subsequently retrained using the new dataset composed of ground-truth tiles labeled by the pathologists, as well as tiles annotated by the model trained in the previous step (Fig. 1d).

Since the weak labels are available at the slide level (patient diagnosis), we remove from the new augmented dataset all impossible labels before retraining (e.g. if the particular tile belongs to a slide with a diagnosis of IDH-wildtype (glioblastoma), possible classes are glioblastoma, normal brain tissue or necrosis; tiles labeled with other classes are removed). This ensured that the model was focused on classes that were contextually relevant for the given WSI, potentially improving its ability to discern subtle differences between relevant categories.

Furthermore, to prevent using the predictions where the model was insecure, we add only the tiles with the predicted class probability larger or equal 90%. Setting a high confidence threshold increases the overall reliability of the pseudo labels, thus reducing noise in the training data.

### 2.4 Performance metrics

For the evaluation of models' performances, we use balanced accuracy, precision, recall and F1-score. While in binary classification balanced accuracy is defined as the arithmetic mean of sensitivity and specificity, for multi-class classification problems it equals the macro-average of recall scores per class. For precision, recall and F1-score macro averaging is used, which reduces the multi-class problem to multiple one-vs-all binary predictions, computes the corresponding performance metric per class, and averages the results over all classes. Macro averaging assumes assigning equal weights to all classes, but class imbalance is handled by weighing the loss function instead. Note that in this case the balanced accuracy is defined in the same way as recall.

## 3 Results

### 3.1 Out-of-domain transfer learning

As a baseline for performance evaluation, we use multiple CNN-based models (VGG16, VGG19, ResNet18, ResNet50, DenseNet121, InceptionV3, MobileNetV2), transformer-based models (ViT, Swin-T) as well as a hybrid CNN-transformer model (ResNet50-ViT) trained from scratch on the in-house dataset. Our aim is to evaluate how much pretraining benefits the performance. The models are carefully selected to: 1) test a variety of conceptually different deep learning architectures; and 2) include architectures that are the same (or comparable) to the ones used in out-of-domain transfer learning.

Before further processing, all images (tiles) are normalized using the in-house dataset statistics, by subtracting the mean and dividing by the standard deviation for each channel. Given that dataset is imbalanced, we use categorical cross-entropy loss weighted by the class weights computed as the inverse class frequency of the labels in the training dataset. For estimating the model performance on the test dataset we use the model weights from the best-performing epoch. PyTorch is used to develop and train deep learning models. Models were trained using the NVIDIA Quadro RTX 6000 GPUs. The results for the baseline deep learning models trained from scratch are provided in Table 5.

**Table 5:** Performance evaluation for glioma subtype classification using out-domain transfer learning (without pretraining and with the models pretrained with ImageNet).

**Pretraining**	**Model**	**Balanced accuracy**	**Precision**	**Recall**	**F1 score**
No pretraining	VGG16	85.07	83.29	85.07	82.53
	VGG19	85.00	83.75	85.00	84.34
	ResNet18	88.22	79.12	88.22	82.38
	ResNet50	78.18	79.49	78.18	78.14
	InceptionV3	79.26	79.78	79.26	79.42
	MobileNetV2	84.54	85.20	84.54	84.59
	DenseNet121	85.10	85.03	85.10	84.53
	ViT	68.25	54.62	68.25	60.09
	Swin-T	57.21	60.51	57.21	58.19
	ResNet50-ViT	81.02	78.34	81.02	77.22

ImageNet	VGG16	94.81	**97.04**	94.81	**95.72**
	VGG19	93.94	94.19	93.94	93.75
	ResNet18	85.89	89.84	85.89	87.23
	ResNet50	92.42	94.65	92.42	93.50
	InceptionV3	85.70	91.02	85.70	87.25
	MobileNetV2	84.70	84.46	84.70	83.45
	DenseNet121	88.97	92.46	88.97	90.26
	ViT	92.38	93.90	92.38	92.98
	Swin-T	86.22	93.82	86.22	89.16
	ResNet50-ViT	**94.87**	96.63	**94.87**	95.48

To evaluate the impact of the out-of-domain transfer learning, we use the same network architectures, but this time the models were pretrained using the ImageNet dataset. To improve the models' generalization ability to a target dataset containing H&E stained images, that deviate substantially from ImageNet, we unfreeze all layers, and fine-tune the models with a small learning rate to avoid overfitting. The results for the baseline deep learning models trained from scratch and using the ImageNet pretrained models are provided in Table 5.

The results in Table 5 show that models pretrained on ImageNet substantially outperform the models trained from scratch on the in-house dataset, with the best-performing model being the hybrid ResNet50-ViT with a balanced accuracy of 94.87%, and F1-score of 95.48%.

### 3.2 In-domain transfer learning

We use 5 models pretrained on large datasets of histopathological images, either using the self-supervised (SimCLR, BYOL, MoCo v3) or the multi-task learning strategy, as explained in Section 2.3.2. To provide a fair comparison, models were trained using exactly the same setup as in out-of-domain transfer learning (see Section 3.1). The obtained results are provided in Table 6, showing that BYOL self-supervised learning with a hybrid CNN/transformer (ResNet50-ViT) backbone reaches the best performance with the balanced accuracy of 96.39% and F1 score of 95.81%.

**Table 6:** Performance evaluation for glioma subtype classification using in-domain transfer learning.

**Pretraining**	**Model**	**Balanced accuracy**	**Precision**	**Recall**	**F1 score**
Self-supervised	SimCLR (ResNet18)	91.57	91.25	91.57	90.60
	BYOL (ResNet50+ViT)	**96.39**	**95.57**	**96.39**	**95.81**
	MoCo v3 (CNN+Swin-T)	93.45	94.65	93.45	93.45

Multi-task	ResNet50	93.15	92.86	93.15	92.54
	DenseNet121	94.31	89.66	94.31	91.43

**Figure 2:**
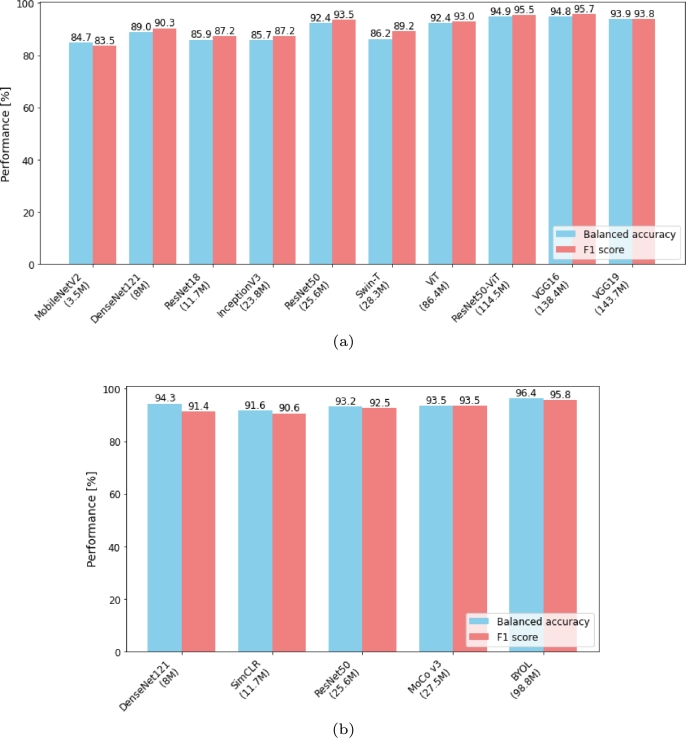
Model performance sorted according to the number of model parameters in ascending order. (a) Out-of-domain transfer learning; (b) In-domain transfer learning.

To allow easier comparison of performance against model complexity, we plot balanced accuracy and F1 score sorted according to the number of parameters in ascending order for out-of-domain transfer learning (Fig. 2a) and in-domain transfer learning (Fig. 2b).

### 3.3 Semi-supervised in-domain transfer learning

**Figure 3:**
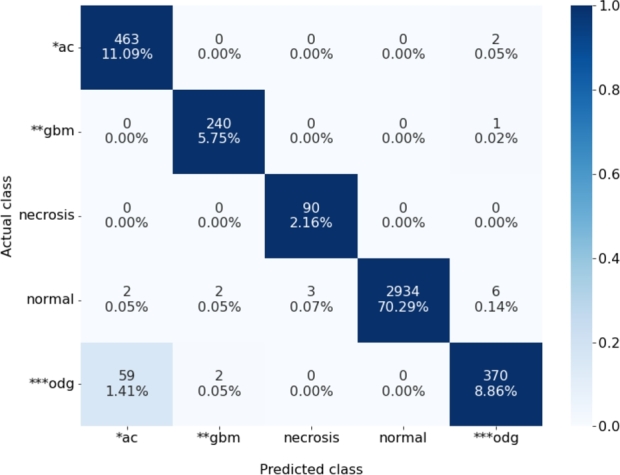
Confusion matrix for the best performing in-domain transfer learning model (semi-supervised ResNet50+ViT). *ac: IDH-mutant (astrocytoma); **gbm: IDH-wildtype (glioblastoma); ***odg: IDH-mutant, 1p/19q codeleted (oligodendroglioma). *Percentages correspond to the fractions of total number of images in the test dataset*.

**Figure 4:**
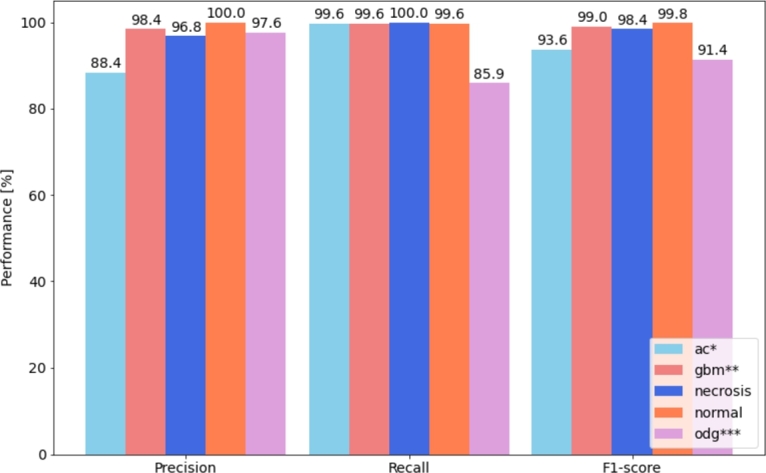
Per class performance for the best performing in-domain transfer learning model (semi-supervised ResNet50+ViT). *ac: IDH-mutant (astrocytoma); **gbm: IDH-wildtype (glioblastoma); ***odg: IDH-mutant, 1p/19q codeleted (oligodendroglioma).

Given the improved performance of the models pretrained in-domain in comparison to out-of-domain transfer learning, we decide to run a two-step semi-supervised learning approach only for the models pretrained in-domain. The summary of results for the in-domain semi-supervised learning is provided in Table 7, whereas confusion matrix and per class performance of the best model (ResNet50 + ViT) are provided in Fig. 3 and Fig. 4, respectively. The performance of the best model reaches balanced accuracy of 96.91% and F1 score of 96.42%.

**Table 7:** Performance evaluation for glioma subtype classification using in-domain transfer learning in a semi-supervised learning scenario.

**Pretraining**	**Model**	**Balanced accuracy**	**Precision**	**Recall**	**F1 score**	**Inference time**
Self-supervised	SimCLR (ResNet18)	96.55	95.27	96.55	95.62	36 s
	BYOL (ResNet50+ViT)	**96.91**	96.22	**96.91**	96.42	64 s
	MoCoV3 (CNN+Swin-T)	96.56	97.03	96.56	96.58	42 s

Multi-task	ResNet50	96.48	**97.94**	96.48	**97.07**	42 s
	DenseNet121	96.63	93.01	96.63	94.57	45 s

We also provide the size of the augmented training datasets after the first step of semi-supervised learning in Table 8 for all in-domain models. Note that the training data size was increased from 2.2 times for SimCLR up to 3.2 times for MoCo v3 in comparison to size of the ground-truth dataset.

**Table 8:** Size of the training dataset after augmentation with semi-supervised labels.

**Type of pretraining**	**Model**	**Dataset size**
Self-supervised learning	SimCLR (ResNet18)	284606
	BYOL (ResNet50+ViT)	416826
	MoCo v3 (CNN+Swin-T)	402347

Multi-task learning	ResNet50	377776
	DenseNet121	339046

### 3.4 Quantitative localization of diffuse gliomas in whole slide images

To aggregate the predictions from the tile level to the WSI level, we overlay the predicted confidence scores for each tile on the WSI as a heatmap, where the red color corresponds to the classes, i.e. 3 diffuse glioma subtypes, normal brain tissue and necrosis, as shown in Fig. 1e. The idea is to draw the pathologist's attention to the most informative areas in the WSI corresponding to the tumor tissue.

**Figure 5:**
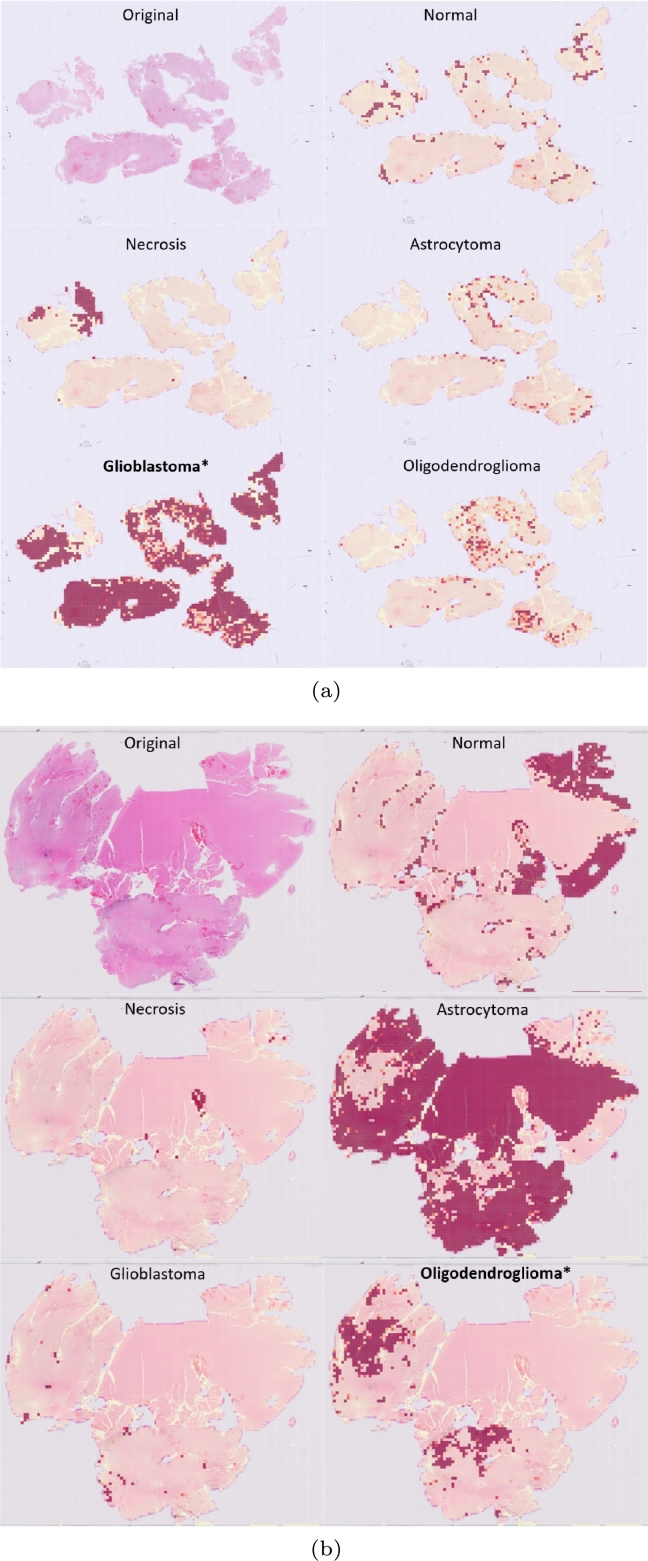
WSI level prediction (semi-supervised ResNet50+ViT). (a) IDH-wildtype (glioblastoma); (b) IDH-mutant, 1p/19q codeleted (oligodendroglioma). *Correct class.

Fig. 5a shows an example of the correct classification of the IDH-wildtype (glioblastoma) tumor using the hybrid semi-supervised ResNet50+ViT model, whereas Fig. 5b presents the case when the model is inconclusive between the IDH-mutant, 1p/19q codeleted (oligodendroglioma) and IDH-mutant (astrocytoma).

We furthermore provide an online tool named DeepHisto freely available to the community (https://bioinfo.lih.lu/deephisto/), where the best performing model (hybrid semi-supervised ResNet50+ViT model) can be used for localization of diffuse gliomas in WSIs.

## 4 Discussion

Out-of-domain transfer learning using the deep neural networks pretrained on ImageNet dataset as “off-the-shelf” feature extractor has become dominant in digital pathology, successfully applied for prediction of tissue types, molecular features, and clinical outcomes [44]. Despite the fact that the models are pretrained in an entirely different domain of natural images, it was shown that models provide satisfactory performance, outperforming the models trained from scratch with limited histopathological image datasets [45]. Another option is to use a model pretrained on ImageNet for weight initialization, and unfreeze the network layers for fine-tuning using the smaller histopathological image dataset, which typically leads to improved performance and faster convergence [46], [47]. Our results in Table 5 confirm these findings, showing substantial performance improvement for both CNN-based and transformer models, that goes up to 32% for Swin-T measured by F1-score. Comparing the results to Table 3 one can observe that smaller models with fewer parameters, such as MobileNet or DenseNet, benefit less from fine-tuning. We furthermore observe that transformer-based models (ViT, Swin-T) reach modest performance in comparison to CNN-based models when trained from scratch, due to the fact that transformers typically require data on a larger scale for pretraining to surpass the lack of inductive biases, such as locality or translational equivariance, which are embedded in CNNs [19]. However, with ImageNet pretraining their performance is comparable to CNN-based models.

Further experiments are done with models entirely trained on a large collection of histopathological image datasets (in-domain transfer learning), either using a semi-supervised approach, or multi-task learning paradigm, as explained in Section 2.3.2. Recent attempts indicate that in-domain transfer learning outperforms out-of-domain transfer learning when sufficiently enough domain-relevant data is available for pretraining [28], [29], [30], [31]. In order to test this hypothesis in a systematic manner, we use all models pretrained on large-scale histopathological data that are available up-to-date (to the best of our knowledge), and fine-tune them under identical conditions on the in-house dataset for the task of prediction of diffuse glioma subtypes. The results presented in Table 6 show improved performance in comparison to models pretrained on ImageNet (Table 5), but to a lower extent than one would expect, given the large mismatch between the domains of natural and histopathological images. Even for the models trained using the same network architectures, there is only a slight improvement of 0.4% in F1-score for the ResNet50-ViT trained with BYOL, around 1% for the DenseNet121 model (trained with multi-task learning), and around 3% for remaining self-supervised learning models, where interestingly for the ResNet50 model (trained with multi-task learning) the performance even slightly drops. This suggests that the learned image features are mostly domain-invariant, requiring only gentle fine-tuning in the target domain with a small learning rate for a limited number of epochs.

Observing the model performance versus the model complexity in Fig. 2, there is a clear trend of performance improvement with the increased number of model parameters, but this is not a linear relationship, and depends also on other parameters, such as e.g. the topological network depth. An example is DenseNet121 with limited number of parameters, but large network depth, resulting in a solid performance with 30% reduced total inference time in comparison to the best performing model, as reported in Table 7. Therefore, opting for a reduced model complexity at the expense of a slight performance decrease is reasonable for deployment in resource-limited conditions.

Analyzing the benefits and challenges related to different in-domain learning strategies, the major advantage of self-supervised learning is that it leverages large amounts of unlabeled data obtained from multiple publicly available datasets, making the most of available resources. Features learned through self-supervised pretraining have demonstrated good transferability to a downstream glioma subtype classification task. On the other hand, self-supervised learning strategies are computationally intensive, which may pose challenges in resource-limited environments or when dealing with large-scale datasets. However, once the models are pretrained, fine-tuning does not require extensive computational resources. Multi-task learning allows the model to jointly learn from multiple related tasks and associated datasets simultaneously, and acts as a form of regularization, preventing overfitting by encouraging shared feature learning across tasks. However, since it requires labeled data, the datasets used for the multi-task learning were substantially smaller than the ones used in self-supervised learning approaches, which may explain the lower relative improvement of multi-task learning than the self-supervised learning, with respect to the out-of domain transfer learning.

Additionally, we want to investigate whether a two-step semi-supervised training, which would augment the initial target dataset with the weakly labeled tiles learned by the model, can boost the model performance even further. Semi-supervised learning improves the performance for all investigated models (see Table 7), with improvements ranging from 0.6% for a hybrid ResNet50-ViT model (pretrained with BYOL), up to 5% for ResNet18 (pretrained with SimCLR).

In order to analyze performance for individual classes we plot in Fig. 3 the confusion matrix of the best performing model (ResNet50-ViT model), concluding that training with the categorical cross-entropy loss function weighted by the class weights has solved the imbalanced data issue, with almost perfect classification for all classes (including the minority classes, such as necrosis), except the IDH-mutant, 1p/19q codeleted (oligodendroglioma) class which is in 16% of cases misclassified as IDH-mutant (astrocytoma). However, due to their morphological similarity, this represents a challenge even for trained pathologists, with commonly confounded diagnoses and large intraobserver variability [48]. Analyzing per class performance in Fig. 4 similar conclusion can be drawn. F1-score is almost perfect for all classes, except astrocytoma and oligodendroglioma. Precision for astrocytoma is approximately 10% lower than for the remaining classes, due to an increased number of false positives (swapping with oligodendroglioma), whereas recall is 15% decreased for oligodendroglioma due to an increased number of false negatives (swapping with astrocytoma). Another interesting observation can be made in Fig. 3: if the model would be used as a binary classifier for tumor detection (class “no tumor” corresponding to normal tissue/necrosis, and “tumor” corresponding to all tumor subtypes), the probability of a false alarm (detecting cancer in normal tissue) is only 0.33%, whereas the probability of misclassification of cancer is equal to 0.

Finally, we generate a slide-level prediction by overlaying the predicted confidence scores for each tile on the WSI as a heatmap, as presented in Fig. 5. Fig. 5a shows an example of the correct classification, where most of the tumor tissue is correctly classified as IDH-wildtype (glioblastoma) using the hybrid semi-supervised ResNet50+ViT model, with only minor incorrectly classified areas of IDH-mutant (astrocytoma) and IDH-mutant 1p/19q codeleted (oligodendroglioma). Necrotic patterns as a hallmark feature of glioblastoma are also correctly recognized, which is of particular diagnostic interest since it correlates with tumor aggressiveness and poor prognosis [49]. However, there are cases where the model is inconclusive and misclassifies areas of IDH-mutant 1p/19q codeleted (oligodendroglioma) as IDH-mutant (astrocytoma), as a result of their morphological similarity (see Fig. 5b).

While the performance results obtained in this study are very high, confirming the potential of using AI in digital pathology, it should be noted that the dataset used for model evaluation is relatively small and may suffer from the limited diversity in terms of number of patients and different scanners being used. Unfortunately, there is a lack of publicly available datasets annotated at the tile level. On the other hand, more diverse WSIs acquired using different scanners from different sources, may also introduce batch effects, where deep learning model tends to learn slide origin, scanner type, or slide preparation (e.g. differences in sample processing and staining), rather than predicting the outcome of interest [50]. However, the primary objective of this study was not to maximize the performance, but to provide a rigorous and fair comparison of various transfer learning strategies for glioma subtype classification from histopathological images. Therefore, limiting potential batch effects might be even preferred in such setup.

## 5 Conclusion

This paper provides a comprehensive experimental analysis of different transfer learning strategies in digital pathology, including in-domain transfer learning, where models are pretrained on medium-to-large-scale publicly available collections of histopathological images, and out-of-domain transfer learning, where models are pretrained on a large collection of natural images (ImageNet). Understanding the nuances of why certain strategies are more effective than the other provides valuable insights into the transferability of features from the pre-trained models. Although in-domain transfer learning provides certain performance improvement, we found that concerns regarding the generalizability of ImageNet representations for the domain of histopathological images are not entirely justified, showing that when fine-tuned properly, out-of-domain transfer learning models can mitigate the impact of limited in-domain data.

The semi-supervised learning approach proposed in this study addresses the constraints posed by limited annotated data, by extrapolating the models beyond the unannotated regions of interest, thus substantially improving the model performance. This not only aids the domain expertise in the diagnostic process, but also effectively reduces the annotation workload for pathologists. The best performing semi-supervised learning model (ResNet50+ViT) achieves balanced accuracy of 96.91% and F1-score of 96.42%.

Finally, we provide a tool for visualization at the WSI level, by generating heatmaps that localize and highlight the tumor areas, therefore drawing pathologist's attention to the most informative areas of the WSI. This adds an interpretability layer to the deep learning models, which is crucial for gaining trust in the model's outputs, and helps the pathologist to understand which regions of WSI contribute more significantly to the model's predictions.

Future work will focus on integrating features extracted from WSIs with molecular profiles extracted from various omics data. Given that molecular profiling techniques can be expensive and not applicable in resource-limited settings, predicting molecular profiles from WSIs offers a cost-effective alternative, utilizing the existing pathology infrastructure and imaging equipment. This approach would allow the extraction of molecular information without the need for additional, often expensive, molecular assays.

## Funding statement

This work was supported by the Luxembourg 10.13039/501100001866National Research Fund (FNR) (C21/BM/15739125/DIOMEDES to P.V.N. and PEARL P16/BM/11192868 grant to M.M.); A-C.H was funded by the Mildred-Scheel Career Center Frankfurt (10.13039/501100005972Deutsche Krebshilfe).

## Ethics statement

The study was reviewed and approved by the National Research Ethics Committee (Comité National d'Ethique de Recherche - CNER), with the approval number REC-LRNO-20110708.

## CRediT authorship contribution statement

**Vladimir Despotovic:** Writing – original draft, Visualization, Validation, Software, Methodology, Conceptualization. **Sang-Yoon Kim:** Writing – review & editing, Visualization, Software, Methodology, Data curation, Conceptualization. **Ann-Christin Hau:** Writing – review & editing, Funding acquisition, Data curation. **Aliaksandra Kakoichankava:** Writing – review & editing, Data curation. **Gilbert Georg Klamminger:** Writing – review & editing, Data curation. **Felix Bruno Kleine Borgmann:** Writing – review & editing, Data curation. **Katrin B.M. Frauenknecht:** Writing – review & editing, Data curation. **Michel Mittelbronn:** Writing – review & editing, Funding acquisition, Data curation. **Petr V. Nazarov:** Writing – review & editing, Methodology, Funding acquisition, Conceptualization.

## Declaration of Competing Interest

The authors declare that they have no known competing financial interests or personal relationships that could have appeared to influence the work reported in this paper.

## Data Availability

Data associated with this study is publicly available in Zenodo (https://zenodo.org/record/7941080). The code and the best performing pretrained models are available at https://git.lih.lu/vdespotovic/deephisto.

## Footnotes

1 https://www.cancer.gov/about-nci/organization/ccg/research/structural-genomics/tcga.

2 http://www.wisepaip.org/paip/.
